# Advances on Prevention and Screening of Gynecologic Tumors: Are We Stepping Forward?

**DOI:** 10.3390/healthcare10091605

**Published:** 2022-08-24

**Authors:** Andrea Giannini, Giorgio Bogani, Enrico Vizza, Vito Chiantera, Antonio Simone Laganà, Ludovico Muzii, Maria Giovanna Salerno, Donatella Caserta, Ottavia D’Oria

**Affiliations:** 1 Department of Medical and Surgical Sciences and Translational Medicine, Sapienza University, 00189 Rome, Italy; 2 Department of Maternal and Child Health and Urological Sciences, Sapienza University of Rome, Policlinico Umberto I, 00185 Rome, Italy; 3 Gynecologic Oncology Unit, Department of Experimental Clinical Oncology, IRCSS-Regina Elena National Cancer Institute, 00144 Rome, Italy; 4 Unit of Gynecologic Oncology, ARNAS “Civico-Di Cristina-Benfratelli”, Department of Health Promotion, Mother and Child Care, Internal Medicine and Medical Specialties (PROMISE), University of Palermo, 90127 Palermo, Italy; 5 Obstetrics and Gynecological Unit, Department of Woman’s and Child’s Health, San Camillo-Forlanini Hospital, 00152 Rome, Italy; 6 Gynecology Division, Department of Medical and Surgical Sciences and Translational Medicine, Sant’Andrea University Hospital, Sapienza University of Rome, Via di Grottarossa 1035, 00189 Rome, Italy

According to 2020 comprehensive global cancer statistics published by the International Agency for Research on Cancer, gynecologic malignancies accounted overall for 16.5% of 8.2 million estimated new cancer cases in women [1]. Gynecological cancers represent an ongoing source of concern due to their high incidence and cancer-related mortality [2,3,4]. 

Although significant therapeutic advances occurred in recent years, patients’ outcomes still remained poor. Indeed, the adoption of innovative treatments (including targeted agents with potential anticancer effects such as antiangiogenic agents, poly (ADP-ribose) polymerase (PARP) inhibitors, tumor-intrinsic signaling pathway inhibitors, and immune checkpoint inhibitors) are still reserved in patients with advanced or recurrent disease [2,3,4,5]. 

There are three approaches used to manage cancers, based on the concept of primary, secondary, and tertiary prevention. The primary prevention approach focuses on preventing disease before it develops (i.e., prophylactic treatment); secondary prevention attempts to detect the disease as early as possible (i.e., early detection); and tertiary prevention is directed at managing a present disease (i.e., treatment) [6,7,8,9].

Cervical cancer represents an ideal model in which to apply primary, secondary, and tertiary preventions. Prophylactic vaccination against HPV aims to reduce the burden of HPV infection and HPV-related lesions (including both precancerous lesions and overt cervical cancer) [6,7]. Vaccination (including bivalent, quadrivalent, and nonavalent vaccines) reduce the incident and persistent infections from HPV types included in the vaccines. Cross protection against other HPV types, not included in the vaccine, is shown only in vaccines that have the adjuvant substance 04 (AS04) [6,7]. Accumulating evidence suggests that the adoption of the vaccines resulted in a significant decrease in HPV infection, HPV lesions, and cervical cancer [6,7,8,9]. In particular, the most recent Cochrane systematic review about the topic clarified that both nonavalent and quadrivalent vaccines offer similar protection against a combined outcome of cervical, vaginal, and vulval precancer lesions or cancer [10].

Secondary prevention (i.e., screening) is widely adopted to prevent cervical cancer. Screening is defined as the identification of early signs of a specific disease in apparently ‘healthy’ people who do not have any symptoms, to provide early detection and to reduce mortality. The adoption of Pap smear and HPV testing resulted in a significant increase in the diagnosis of cervical dysplasia and a significant decrease in cervical cancer. Moreover, mobile health interventions effectively improve cancer screening rates in the general population, but they are necessary for the poorest countries. New screening methods, such as p16/Ki67 [11], HPV self-testing, and the use of artificial intelligence in colposcopic assessment, should be disseminated [7,8]. It is useful to focus on precancerous lesions in order to create tools for women regarding their risk of persistence/recurrence after primary conization and identify categories at higher risk compared with the other ones [9]. Tertiary prevention in cervical cancer includes its treatment with surgery (radical hysterectomy), radiotherapy, and systemic treatments (chemotherapy, bevacizumab, and immunotherapy) [12]. This can be considered of paramount importance, since a recent systematic review and meta-analysis found that women treated for cervical intraepithelial neoplasia (CIN) have a considerably higher risk of later being diagnosed with cervical and other HPV-related cancers compared with the general population: in particular, the higher risk of cervical cancer lasts for at least 20 years after treatment and is higher for women of more than 50 years of age [13]. In this context, a significant risk reduction of developing recurrent CIN after surgical excision and HPV vaccination was shown compared to surgical excision only [14]. Since cervical cancer is a predictable disease, we need more efforts to implement prevention, screening, and early diagnosis in cervical cancer.

Unfortunately, the implementation of primary and secondary prevention in other gynecological cancers is not optimal at present.

Endometrial cancer occurs in postmenopausal women with an average age at diagnosis of 60 years, and the risk factors associated with this tumor are widely known [15]. Although there is no standardized screening test for endometrial cancer, prevention is possible following healthy behaviors (prevention of obesity, weight gain, metabolic syndrome, and diabetes). As confirmed by a recent systematic review and meta-analysis, early detection strategies focused on women with post-menopausal bleeding have the potential to capture as many as 90% of endometrial cancers [16].

Furthermore, the identification of women with hyperplasia and atypia is of paramount importance to reduce the burden of endometrial cancer. The identification of women suffering with abnormal uterine bleeding, and with those with abnormal endometrial thickness (evaluated by trans vaginal ultrasound (TVS)) [17] is making screening and early diagnosis possible, even in endometrial cancer patients [18]. Finding a screening test for endometrial cancer is challenging, but early detection is more and more possible. This should be considered a research priority, since a recent meta-analysis of prospective studies found that adherence to cancer prevention guidelines was negatively related to endometrial cancer risk [19].

TVS screening for endometrial cancer has good sensitivity in postmenopausal women, as shown by a case–control study within the United Kingdom Collaborative Trial of Ovarian Cancer Screening (UKCTOCS) cohort [20]. Recent studies have shown that urine CA125 displays potential as a diagnostic marker for symptomatic women with suspected endometrial cancer [21], but more robust molecular markers are still to be implemented in the clinical practice [22].

Ovarian cancer is the deadliest gynecologic cancer, despite the incidence of this tumor being relatively low. Currently, ovarian cancer is a target of intense research because it is often not discovered until the disease is advanced, which causes significant mortality [23]. Screening is not currently recommended in the general population, and some countries offer surveillance of high-risk women, with specific genetic patterns and positive family history, with a lifetime ovarian cancer risk of 10% or more [24].

CA125 is the typical biomarker of serous ovarian cancer used in recent decades. The application of this tumor biomarker has changed using longitudinal algorithms in recent years, trying to be more effective [25]. In addition, pooled data from a recent and well-designed meta-analysis confirmed the overall diagnostic value of Serum Human Epididymis Protein 4 (HE4) in ovarian cancer [26]. In addition to these biomarkers, accumulating evidence from other meta-analyses suggests that future strategies for early detection of ovarian cancer may be based on non-invasive evaluation of circulating tumor DNA [27], as well as circulating microRNA profiling [28,29].

The randomized controlled trial UK Collaborative Trial of Ovarian Cancer Screening (UKCTOCS) has shown that the use of longitudinal CA125-based multimodal screening (MMS), or annual TVS (USS) is not useful for reducing mortality for OC. However, the number of women affected by low-volume invasive epithelial ovarian and peritoneal cancer (stage I, II, and IIIa) was higher in the MMS group than in the ultrasound counterpart [30]. The Prostate, Lung, Colorectal, and Ovarian Cancer Screening Trial (PLCO) confirmed that there was no difference in number of deaths for OC between the screen and no screen arms, analyzing 78,286 women at a median follow-up of 14.7 years [31]. In the same way, UK FOCSS Phase I had the same results, showing that annual screening is ineffective for the general population when it comes to detecting early stage disease [32]. Although no screening strategies have been identified to definitively decrease ovarian cancer mortality at the moment, a strategy based on the longitudinal CA125 profile and second-line TVS performed by skilled operators can guide to earlier diagnosis of OC in the general population, not only in the high-risk group. Notably, The US Food and Drugs Administration (FDA) highlighted that “Health professionals should not recommend ovarian cancer screening tests to women who do not have any symptoms because of the high possibility of unreliable results” and, more importantly, that “there are currently no screening tests for ovarian cancer that are sensitive enough to reliably screen for ovarian cancer” [33].

In this context, are screening techniques and prevention strategies for gynecologic cancers adequate? For cervical cancer, effective screening methods exist, although they are underused. For endometrial and ovarian cancers, no effective screening methods exist.

There is an urgent need to identify novel strategies to detect all gynecologic tumors as early as possible, thus reducing mortality and improving the quality of care.
